# Development, validation and initial evaluation of patient-decision aid (SUI-PDA©) for women considering stress urinary incontinence surgery

**DOI:** 10.1007/s00192-019-04047-z

**Published:** 2019-08-03

**Authors:** Hui Ling Ong, Inna Sokolova, Holly Bekarma, Claire Curtis, Alastair Macdonald, Wael Agur, on behalf of NHS Ayrshire & Arran Continence Multidisciplinary Team

**Affiliations:** 1 University of Glasgow, Glasgow, UK; 2 University Hospital Crosshouse, NHS Ayrshire & Arran, Kilmarnock, UK; 3 University Hospital Ayr, NHS Ayrshire & Arran, Ayr, UK; 4 Person-Centred Health and Care Programme, Healthcare Improvement Scotland, Edinburgh, UK; 5 Glasgow School of Art, Glasgow, UK

**Keywords:** Decisional conflict scale, Patient decision aid, Stress urinary incontinence, Surgery

## Abstract

**Introduction and hypothesis:**

Following the design, face validation and publication of a novel PDA for women considering SUI surgery, the main objective of the study is to evaluate the usefulness of SUI-PDA© by using a validated tool to obtain patient feedback.

**Methods:**

From July 2018 to March 2019, the PDA, already incorporated into the patient care pathway, was objectively evaluated using the Decisional Conflict Scale (DCS) to determine patients’ views. Patients recorded their values and reasons for requests and declines of treatment. The total DCS score, scores from each DCS subgroup and individual patient responses were calculated and analysed.

**Results:**

The mean age of the first 20 patients to complete the DCS was 54 years, the mean BMI was 30.1 and the median parity was 3. The average total DCS score was only 9.29 out of 100 (range 0–29.69) suggesting that the PDA was quite useful for patients considering SUI surgery. Overall, the PDA had largely favourable responses across all five DCS subgroups. The ‘informed’ subgroup had the best score (6.67) while the ‘uncertainty’ subgroup had the least favourable score (14.58). Despite the procedure pause, the mesh tape option remained on the PDA; however, no patient had chosen this option, with a large proportion citing ‘*safety’* issues as the main reason. Bulking agent injections were the most popular choice (40.0%) and the most commonly performed procedures (50.0%) mainly because of quicker ‘*recovery’*. The second most popular participant choice was colposuspension (35.0%) followed by autologous fascial sling (25.0%), with women citing ‘*efficacy’* as the main reason behind their choice.

**Conclusion:**

SUI-PDA© was reported by patients and clinicians to be useful with clinical decision-making for SUI surgery. Further validation in a larger patient group is underway.

**Electronic supplementary material:**

The online version of this article (10.1007/s00192-019-04047-z) contains supplementary material, which is available to authorized users.

## Introduction

SUI is the loss of urine when coughing, laughing, sneezing or exercising. It is a common and distressing condition, with negative impact on quality of life. The prevalence varies from 20 to 50% and is associated with age and parity [1]. If conservative treatment, e.g. pelvic floor muscle training, is not successful, the most successful surgical procedures are mid-urethral mesh tape, colposuspension, autologous fascial sling and urethral bulking agent injections. Women have a 10% lifetime risk of requiring continence surgery [1].

Between April 2008 to March 2017, procedure data from the UK NHS confirmed that 100,516 patients had a mid-urethral tape procedure, while only 1195 patients had a non-tape SUI procedure [2]. Although the 2013 national guideline from The National Institute for Health and Care Excellence (NICE) recommended that tape and non-tape SUI procedures be offered equally [3], 84 mesh tape procedures were performed for every 1 non-tape procedure over the 10-year period [2]. Hundreds of patients recently engaged in litigation on the basis of lack of informed consent, particularly in offering alternatives to the mesh tape option. In July 2018, the safety concerns led to a pause in the use of vaginal mesh in The British Isles and prompted an increasing desire to explore the non-mesh alternatives.

Little is known, however, about how patients choose among different treatment options for SUI [4] and there are no validated patient decision aids (PDAs) in this context.

PDAs have been shown to increase patient knowledge, clarity about their own values and accuracy of risk perceptions regarding various management options [5]. Women considering SUI surgery require up-to-date information on all common and available surgical procedures as well as support in their decision-making, tailored to their values and needs.

Our team designed and developed a novel SUI surgery patient decision aid (SUI-PDA) to help women in making a choice of treatment based on their own individual values. This study reports the development and validation of SUI-PDA as well as the initial evaluation of its usefulness in clinical practice for women considering SUI surgery.

## Methods and materials

**Fig. 1:**
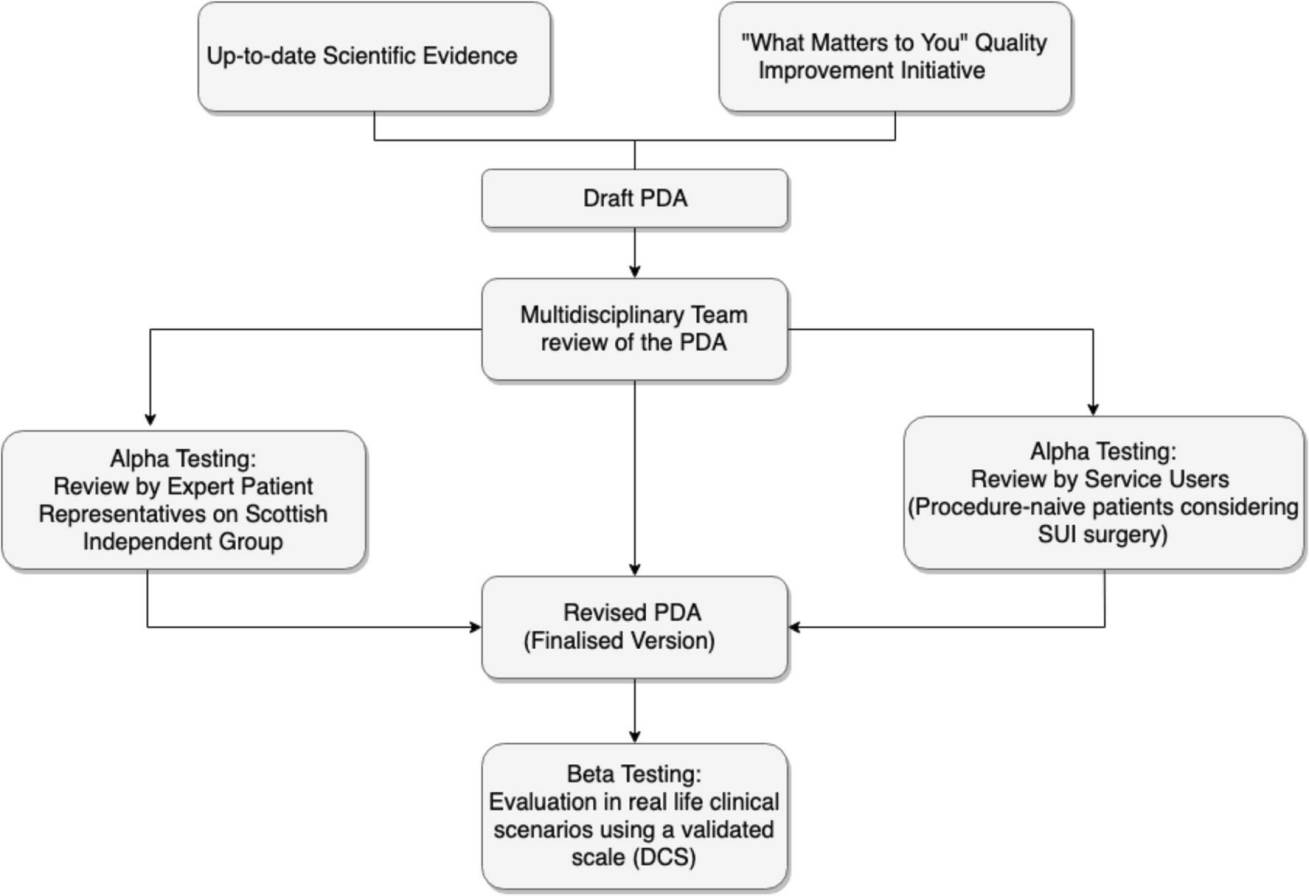
SUI-PDA development flowchart

The Methods and Results section follows the SUNDAE Statement (Standards for UNiversal reporting of patient Decision Aid Evaluation studies) [6] of conducting and reporting studies evaluating PDAs in clinical settings (Fig. 1).

### Face validation

The ‘literature review’ stream of the relevant Scottish Government Independent Group, published in 2015 [7], provided the scientific evidence base upon which the information within SUI-PDA© were populated and represented. The early design followed the broad lines of the *‘What Matters to You’* Initiative [8] of NHS Scotland to encourage patients to explore their own values before making their decision. The first version was drafted and face-validated by members of NHS Ayrshire & Arran Continence Multi-Disciplinary Team (MDT), including urogynaecologists, urologists, physiotherapists, continence specialist nurses and a clinical librarian. The PDA was later reviewed by members of the relevant Scottish Government Expert Group including expert patient representatives (who had good outcomes and those who had adverse outcomes of continence surgery), an improvement adviser from the NHS Scotland Person-Centred Department and an academic in design from Glasgow School of Art. Concomitantly, face validation continued with service users, the procedure-naïve patients considering SUI surgery. The comments were fed back to the core development team and eight versions of the four components of the PDA were produced following team discussions—including two meetings in person as well as group email communications. Plain English guidelines were followed to produce clear and concise patient information to communicate complex medical terminology in a language that is easy to understand. The final version of the SUI-PDA was published on the NHS Ayrshire & Arran website [8] on 3 November 2017.

### Components of SUI-PDA

**Fig. 2:**
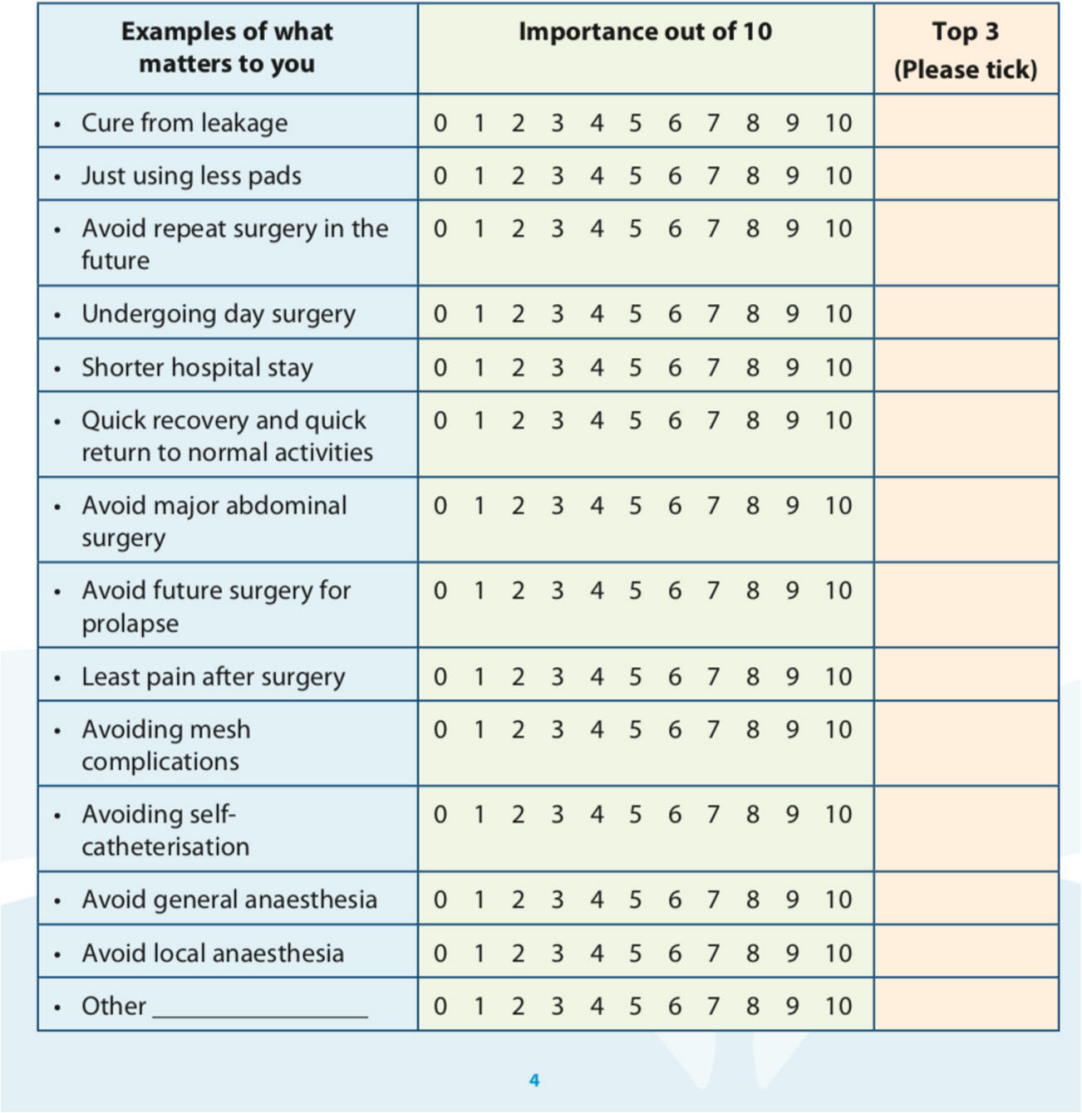
‘What matters to me’ component of SUI-PDA

**Fig. 3:**
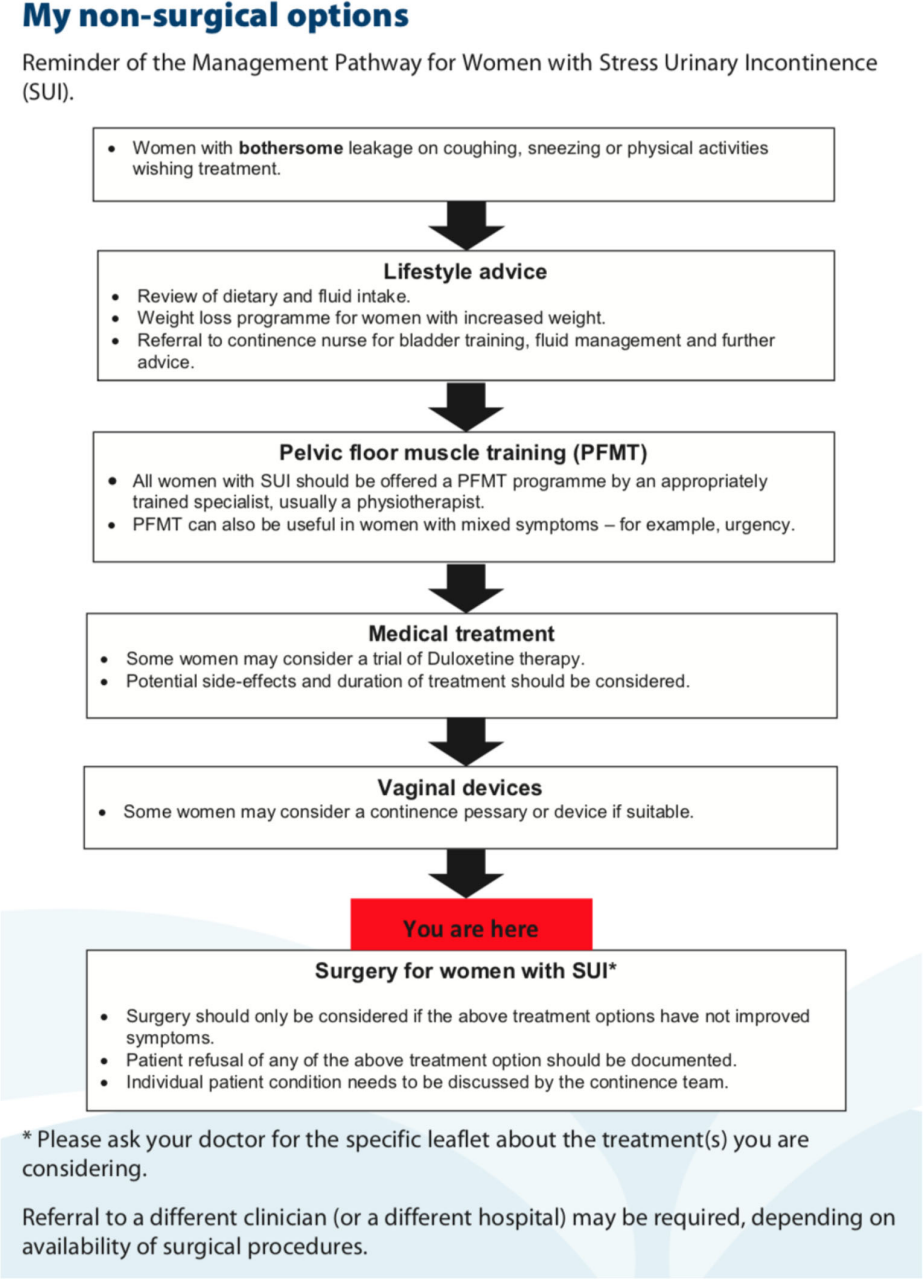
‘Treatment pathway’ component of SUI-PDA

The SUI-PDA is a purpose-built, decision-support booklet structured to guide the patient through a sequence of four components: *What Matters To Me*, *Care Pathway*, *Procedure Comparison* and *Request-for-Treatment*. The ‘*What Matters To Me*’ component (Fig. 2) clarifies patients’ own values and is comprised of 13 aspects of clinical care that may matter to women considering SUI surgery, e.g. *‘Avoid major abdominal surgery’* and *‘Quicker recovery*…’. Patients were asked to rate every aspect on a 11-point visual analogue scale (VAS) and select the three most important to them. A free text box was provided to capture any other aspect of care, unique to the individual. The ‘*Care Pathway’* [Fig. 3] reminds the patients of the various non-surgical treatment options, highlights their position on the pathway and justifies the need for surgery at the point of decision-making.

**Fig. 4:**
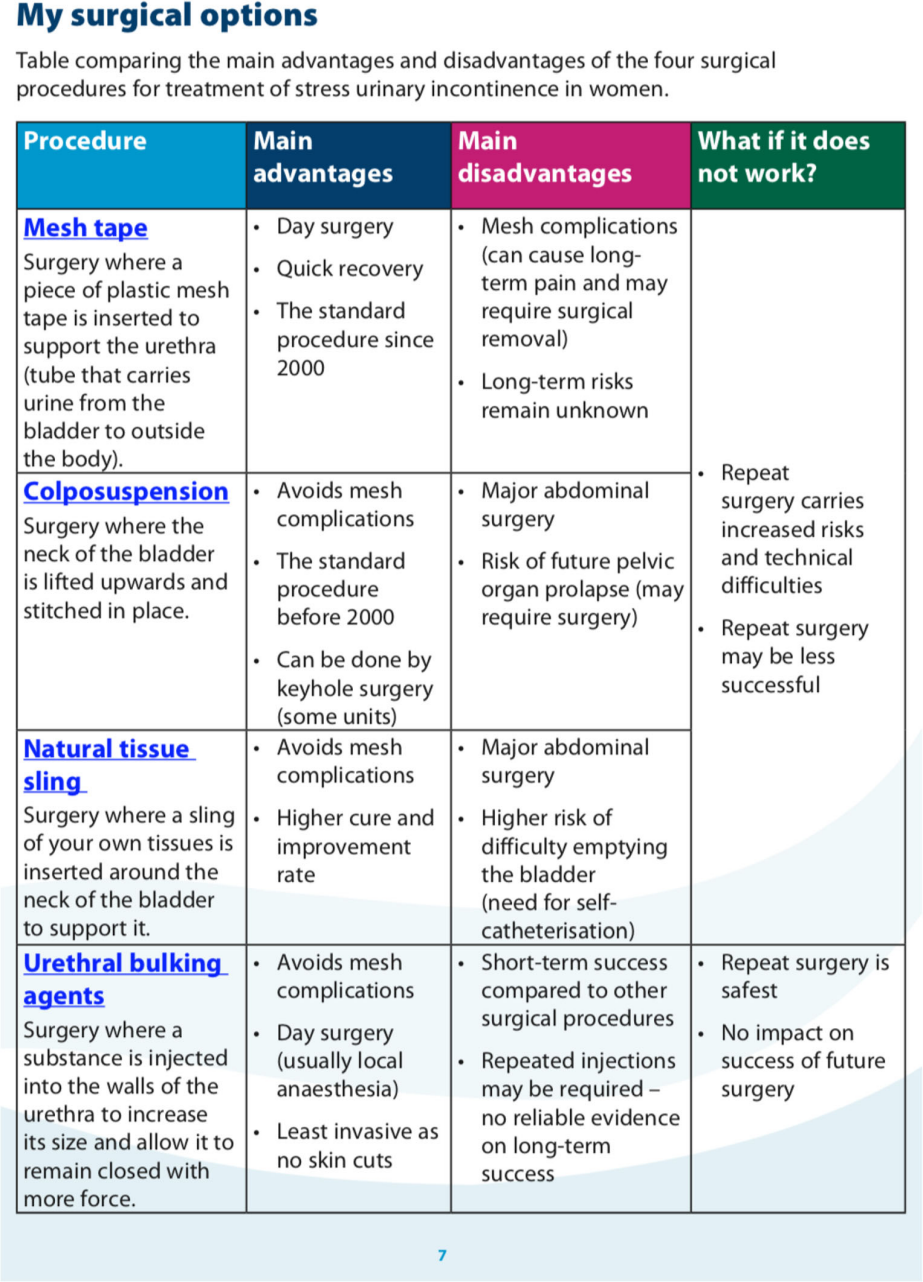
‘Procedure comparison’ component of SUI-PDA

**Fig. 5:**
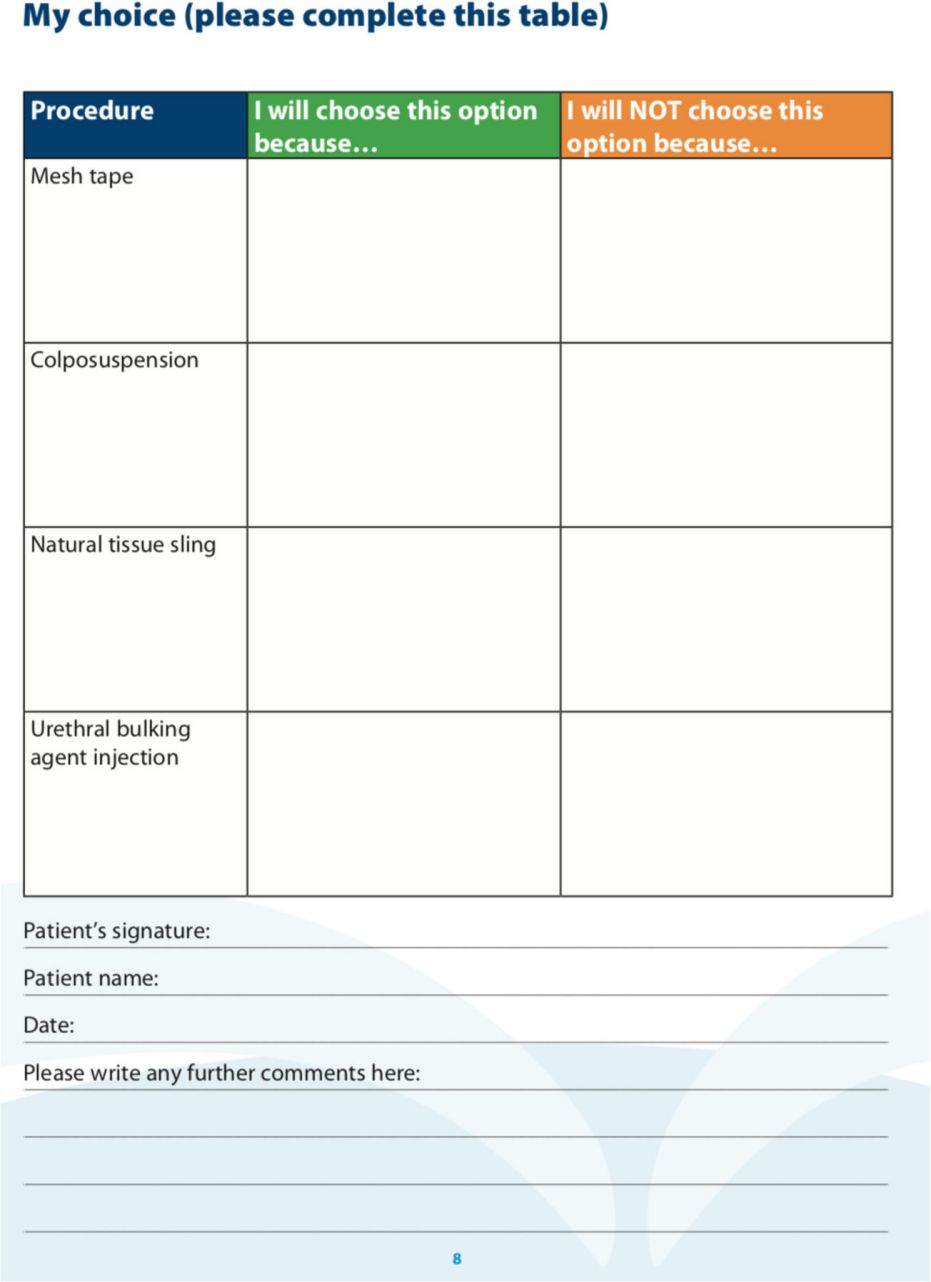
‘Request for Treatment’ component of SUI-PDA

The ‘*Procedure Comparison’* component (Fig. 4) summarises the information on the four main surgical treatment options (mesh tape, colposuspension, autologous sling and bulking agent injection) into tables. The main risks and benefits of each treatment option are clearly outlined to ensure that patients can visually compare the differences. The development team has reached consensus on the main advantages and disadvantages following the literature review [9–13]. Due to the wide variation in the risk incidences reported in the literature for the four procedures, the group decided not to include any specific figures on adverse events. This approach is consistent with the one taken by the various UK groups developing national patient information leaflets [14]. The comparison table also provided information on what happens if each surgical option does not work in controlling urine leakage. The last component, ‘*Request for Treatment’* (Fig. 5), is interactive as the patient requests one of the four procedures and provides reason(s). This component also asks for reasons behind declining the other three options. The reasons behind the requests for and declines of treatment options were categorised into four themes: ‘efficacy’, ‘safety’, ‘invasiveness’ and ‘recovery’.

### Delivery of the SUI-PDA

All women had completed a conservative treatment programme, had urodynamics-proven SUI and were considering SUI surgery in either the Urology or Gynaecology Department. SUI-PDA is not a stand-alone tool, but is rather the final stage of decision-making after patients have read the updated national leaflets for the four SUI procedures published by The Scottish Government, The British Association of Urological Surgeons (BAUS) and The British Society of Urogynaecology (BSUG).

The SUI-PDA was delivered to all patients by the specialist nurses who performed their urodynamics studies. The sample was sequential, not random, as all women spoke English as first language and completing the PDA is an essential criterion before discussion at the MDT meeting and subsequent surgery. Therefore, all women were asked to complete the SUI-PDA prior to being informed of availability of individual options. While the two hospitals had followed the mesh pause, the synthetic mid-urethral tape procedure remained as one of the options on the PDA—with a clear plan for external referral if a woman requested it. At the point of making a choice of which SUI procedure to go for, and before consultations with the surgeons, patients were asked to read and complete the SUI-PDA. Patients were specifically asked to (1) indicate their three most important values using a visual analogue scale (0 = not important, 10 = most important), (2) request only one surgical procedure to undergo, (3) provide their own reason(s) behind their choice and (4) evaluate the usefulness of the PDA by completing the Decisional Conflict Scale (DCS). The completed SUI-PDA was filed in patient records prior to consultation with the surgeon. Subsequently, the Continence Multi-Disciplinary Team (MDT) reviewed individual patient’s clinical condition, previous interventions, individual values and procedure choice on the SUI-PDA before a group clinical decision was made.

Responses collected from SUI-PDAs and DCSs were anonymised and data were entered into a secure database prior to statistical analysis. All calculations were made using SPSS Statistics (version 23). The evaluation project did not require research ethics approval. The confirmatory letter from the Research and Development department, NHS Ayrshire and Arran, is in Appendix B.

## Results

The delivery of SUI-PDA to women considering SUI surgery in the two hospitals of our Health Board, University Hospital Crosshouse and University Hospital Ayr, had high fidelity. The SUI-PDA has been incorporated into the continence care pathway (Fig. 3) and its completion had become an essential component of the paperwork and a requirement prior to MDT discussion. Although 'doing nothing' was an available option on the pathway, all women who completed the PDA decided to go ahead with surgery. At the time of writing, 40 women have completed the PDA and 20 have completed the evaluation using the DCS.

### Impact on service

The use of SUI-PDA has significantly impacted the service and the pattern of continence surgery in our hospital. One example of positive influence is our responsiveness to patient choice by re-introducing the autologous fascial sling procedure in our hospital in spring 2016 [15]. However, one patient expressed negative hindsight by stating her wish that this PDA had been available prior to her first continence surgery. She believed that had she been given the choice among the four procedures, she would not have agreed to undergo the one that had left her with long-term complications.

### Usefulness in patient choice and decision-making

From July 2018 to March 2019, this evaluation project collected data for the first group of women who completed the SUI-PDA and the Decisional Conflict Scale (DCS). The DCS (Appendix A) is the validated and the most commonly used tool in evaluating the usefulness of PDAs to patients [16]. It comprises a 16-item statement format, categorised into 5 subgroups (*uncertainty, informed, values clarity, support* and *effective*) with 5 response category statements (*strongly agree, agree, neither agree nor disagree, disagree and strongly disagree*). A total score was calculated per patient as well as the individual score for each of the five subgroups. A total score of 0 means ‘no decisional conflict’ and a score of 100 means ‘extremely high decisional conflict’.

Forty women have completed the PDA since the start date in September 2016, and 20 women completed both the PDA and DCS from July 2018 to March 2019. The average age for those who completed both SUI-PDA and DCS was 54 years, the average BMI was 30.1 and the median parity was 3. All women were white Caucasian and English was their first language.

Table 1 shows the frequency of requests and declines for treatment. Bulking agent injections were the most common choice of procedure (8/20, 40%), and their most common acceptance theme was *‘recovery’*, followed by colposuspension (7/20, 35.0%) and autologous sling (5/20, 25%). Although the option of using the mesh remained on the PDA, no patient had chosen this option. The most common reason for decline conformed to the theme *‘safety’*. It was difficult to unpack a true understanding of the mesh-related adverse events mentioned in the PDA from the effect of the negative stories in the media. However, seven women mentioned the ‘*media’* attention in their stated reason behind declining the mesh tape option. Three women declined the mesh option as being ‘too invasive’, ‘invasive and risky’ and ‘big operation’. Surgical choice did not appear to be influenced by age, BMI or parity. Table 2 shows that cure from leakage, avoiding repeat surgery and quick recovery, were the top three values/concerns indicated by patients. Table 3 relates the procedure performed to the corresponding top three values/concerns indicated by patients.

**Table 1:** Frequency of patients’ requests and declines for treatment

	No. of request for treatment (%)	Commonest request theme	No. of decline of treatment (%)	Commonest decline theme	MDT Choice no.(%)^a^
Mesh tape	0 (0.0)	–	20 (100)	Safety (*n* = 13, mesh complications)	0 (0)
Colposuspension	7 (35.0)	Efficacy	13 (65.0)	Recovery (*n* = 4)	5^b^ (25.0
Autologous fascial sling	5 (25.0)	Efficacy	15 (75.0)	Safety (*n* = 10, self-catheterisation)	5 (25.0)
Bulking agent injections	8 (40.0)	Recovery	12 (60.0)	(Lack of) efficacy (*n* = 12)	10 (50.0)

^a^All performed procedures matched MDT choice

^b^One patient had later agreed to undergo the procedure suggested by the MDT (bulking agent injection) rather than her original choice on the PDA (colposuspension)

**Table 2:** Frequency of selected top three values/concerns by patients

Value/concern	No. of patients choosing value in top 3 choice (%)
Cure from leakage	18 (90.0)
Avoid repeat surgery in the future	10 (50.0)
Quick recovery and return to normal activities	7 (35.0)
Avoiding mesh complications	6 (30.0)
Avoiding self-catheterisation	6 (30.0)
Just using fewer pads	2 (10.0)
Undergoing day surgery	1 (5.0)
Avoid major abdominal surgery	1 (5.0)
Avoid future surgery for prolapse	3 (15.0)
Least pain after surgery	1 (5.0)
Avoid general anaesthesia	1 (5.0)

**Table 3:** Frequency of procedure performed and the corresponding top three values/concerns indicated by patients

	Cure from leakage	Avoid repeat surgery	Quick recovery and quick return to normal activities	Avoid self-catheterisation
Mesh	0 (0)	0 (0)	0 (0)	0 (0)
Colposuspension	7 (38.9)	4 (40.0)	3 (42.9)	3 (50.0)
Autologous fascial sling	5 (27.8)	3 (30.0)	1 (14.2)	1 (16.7)
Bulking agent injections	6 (33.3)	3 (30.0)	3 (42.9)	2 (33.3)

The total DCS score was 9.29 (range 0.0 – 29.69), suggesting an overall usefulness of the SUI-PDA to women considering surgery (Table 4). Women found the *Information* domain to be the most helpful (6.67), whereas the relatively higher score for *Uncertainty* (14.58) suggests that, despite using the SUI-PDA, some women still lacked full confidence in their choices.

**Table 4:** Decisional Conflict Scale (DCS) scores

DCS subgroup (no. of patients)	Mean score^a^ (SD)	Range
Informed (*n* = 20)	6.67 (10.68)	0.00–33.33
Values (*n* = 19)	10.53 (10.70)	0.00–25.00
Support (n = 19)	7.89 (11.61)	0.00–33.33
Uncertainty (n = 20)	14.58 (18.71)	0.00–58.33
Effective (n = 19)	8.88 (13.23)	0.00–43.75
Overall DCS Score (n = 19)	9.29 (9.29)	0.00–29.69

^a^Score ranges from 0 to 100, zero (0) meaning no decisional conflict and 100 meaning strong confliction

There was one instance of disagreement between patient choice and MDT choice. The patient had requested a colposuspension procedure but later agreed with the MDT view to undergo, at least initially, the less invasive bulking agent injection because of co-morbidities. However, her individual total DCS score remained low at 4.89, suggesting overall confidence and usefulness of the PDA to her original decision-making.

## Discussion

### Principle statements

This is the first study in the scientific literature to report the development, validation and initial evaluation of a patient decision aid for women considering surgery for stress urinary incontinence in a real-life clinical practice. The four-component SUI-PDA worked as intended and appears to be useful in assisting women considering SUI surgery by (1) clarifying their values, (2) providing information on individual procedure’s benefits and risks and (3) direct support and reasoning during the decision-making process.

The PDA provided valuable insight into how women make surgery choices which mostly, but not always, were based on their pre-expressed values. For example, it demonstrated how the majority of women had prioritised safety over efficacy by choosing to undergo a bulking agent procedure as their first choice. This is consistent with what Robinson et al. had suggested in “What do women want?” [4]. Another example was the apparent difference in perception of ‘*invasiveness’* between clinicians and women. Although the tape procedures were described in the literature as *minimally invasive*, three women declined this option and indicated (on the Request-for-Treatment section) a perception that the permanent mesh device is ‘too invasive’, ‘invasive and risky’ and a ‘big operation’.

For the first time, our study suggests that our robustly designed PDA is useful for women considering SUI surgery and has the potential to achieve similar benefits shown by PDAs in other clinical conditions [5]. The Cochrane systematic review of a wide range of conditions has shown that PDAs decreased decisional conflict related to patients feeling uninformed and decreased the number of patients who were considered ‘passive’ in decision-making [5].

### Relation to other literature

Although no studies in the literature validated a PDA for SUI surgery, there was one attempt to explore the usefulness of a purpose-designed PDA for prolapse surgery [17]. The prolapse PDA, however, was not highly successful as the tool was not validated and did not include a full exploration of individual patient values.

### Clinical implications

The concept of shared-decision-making using Patient Decision Aids is relatively new in the urogynaecology and female urology subspecialty. Although *safety* and *efficacy* have been our primary outcomes for several decades, it is perhaps time to consider *shared decision-making* as our third outcome that is more likely to be achieved with the vast majority of patients [18].

While the 2015 Montgomery ruling [19] is believed by many to have resulted in an increased consultation time for women considering SUI surgery, our PDA has the potential to focus the consultation time and provide the best value. This potential is dependant on the point of introduction into the continence care pathway. In our experience, the best stage of administering the SUI-PDA was at the point of confirming a diagnosis of urodynamic stress incontinence (USI) and prior to discussion with the surgeons or with other MDT members. More data on the cost-effectiveness of using PDAs in general are required, as the Cochrane review did not find that their use decreased healthcare costs and, in some studies, there are in fact increased healthcare costs.

### Strengths and limitations

Our study followed a robust face-validation process that involved all relevant multidisciplinary stakeholders, including patients, using the most robust 16-item evaluation tool, the DCS, and the most comprehensive reporting according to the SUNDAE statement. The SUI-PDA scored highly overall, particularly in the *Information* domain. However, some women remained unsure about their choice, with the least favourable score in the *Uncertain* domain.

Our initial evaluation reports on a relatively small number of women considering SUI surgery. To ensure that our findings are generalisable to a larger population, data collection is still on-going to further validate the usefulness of SUI-PDA in a larger group. As the SUI-PDA had already been incorporated into the continence care pathway, this study could not detect any change in our patients’ decisional conflict before and after introduction of the PDA.

While the mid-urethral tape option was not available in our hospital, there were clear plans for onward referral if a patient chose this option. We believe the media may have influenced the decision of some of our patients; however, the 2014 mesh procedures suspension in Scotland in itself may not have significantly influenced the results of our PDA evaluation project conducted in 2018-2019. Our results can only be applicable to white Caucasian English-speaking women; however, the SUI-PDA is available in other languages by request to our Health Board [8].

### Further research

Further evaluation of the SUI-PDA is underway to consolidate the above findings and to further explore the reasons behind the relatively less favourable score in the *Uncertain* domain. To measure a potential reduction in decisional conflict, a future research project would include a prospective comparative study, preferably randomised, between women who used the PDA and those who engaged only in standard counselling, provided ethical matters are fully considered. In addition, we are also planning to relate the patient choice using SUI-PDA to the outcome of surgery and to study postoperative patient reflections.

## Conclusion

The SUI-PDA was reported by patients and clinicians to be useful with clinical decision-making for SUI surgery. Further validation in a larger patient group is underway.

## Electronic supplementary material

ESM 1 (PDF 81 kb)

ESM 2 (PDF 659 kb)
