# Supplementary material for: Development, validation and initial evaluation of patient-decision aid (SUI-PDA©) for women considering stress urinary incontinence surgery
Source: Int Urogynecol J. 2019 Aug 3;30(12):2013–22. doi: 10.1007/s00192-019-04047-z (PMC6861540; doi:10.1007/s00192-019-04047-z)
Supplement: Supplementary file 1 — (PDF 81 kb) [file 192_2019_4047_MOESM1_ESM.pdf]

My difficulty in making this choice

A. Which surgical option do you prefer? Please check ☑ one and indicate your 2nd, 3rd and last option.

- ☐ **Mesh Tape**
- ☐ **Colposuspension**
- ☐ **Autologous Fascial Sling**
- ☐ **Bulking Agent Injection**
- ☐ **Unsure**

B. Considering the option you prefer, please answer the following questions:

	**Strongly Agree** [0]	**Agree** [1]	**Neither Agree Or Disagree** [2]	**Disagree** [3]	**Strongly Disagree** [4]
1. I know which options are available to me.	☐	☐	☐	☐	☐
2. I know the benefits of each option.	☐	☐	☐	☐	☐
3. I know the risks and side effects of each option.	☐	☐	☐	☐	☐
4. I am clear about which benefits matter most to me.	☐	☐	☐	☐	☐
5. I am clear about which risks & side effects matter most.	☐	☐	☐	☐	☐
6. I am clear about which is more important to me (the benefits or the risks and side effects).	☐	☐	☐	☐	☐
7. I have enough support from others to make a choice.	☐	☐	☐	☐	☐
8. I am choosing without pressure from others.	☐	☐	☐	☐	☐
9. I have enough advice to make a choice.	☐	☐	☐	☐	☐
10. I am clear about the best choice for me.	☐	☐	☐	☐	☐
11. I feel sure about what to choose.	☐	☐	☐	☐	☐
12. This decision is easy for me to make.	☐	☐	☐	☐	☐
13. I feel I have made an informed choice.	☐	☐	☐	☐	☐
14. My decision shows what is important to me.	☐	☐	☐	☐	☐
15. I expect to stick with my decision.	☐	☐	☐	☐	☐
16. I am satisfied with my decision.	☐	☐	☐	☐	☐

Please provide any further information here.

Decisional Conflict Scale. © 1993 [updated 2010]. Available from www.ohri.ca/decisionaid.
Decisional Conflict Scale © AM O'Connor, 1993, revised 2005
