# Supplementary material for: Development, validation and initial evaluation of patient-decision aid (SUI-PDA©) for women considering stress urinary incontinence surgery
Source: Int Urogynecol J. 2019 Aug 3;30(12):2013–22. doi: 10.1007/s00192-019-04047-z (PMC6861540; doi:10.1007/s00192-019-04047-z)
Supplement: Supplementary file 2 — (PDF 659 kb) [file 192_2019_4047_MOESM2_ESM.pdf]

Patient Label

NHS Ayrshire & Arran

What matters to you when choosing surgery for stress urinary incontinence?

Shared-decision tool for patients

Follow us on Twitter @NHSaaa

Find us on Facebook at www.facebook.com/nhsaaa

Visit our website: www.nhsaaa.net

All our publications are available in other formats

Please complete this form and hand back to a member of staff or post to:

Gynaecology secretary
University Hospital Crosshouse
Kilmarnock Road
Kilmarnock
KA2 0BE

Contents **Page**

Contents	Page
A) Why complete this form?	3
B) My values - What matters to me? **To complete**	4
C) My non-surgical alternatives	6
D) My surgical options	7
E) My choice **To complete**	8

Why complete this form?

Introduction

We are working to improve person-centred care for women considering surgery for stress urinary incontinence. It is important that the type of surgery you choose is personalised. As well as being safe and effective, surgery will focus on your individual needs and preferences as much as possible.

We know that by finding out a bit more about you, we can improve shared-decision making and therefore the overall outcome of surgery. One of the ways to make this better is for the doctor/surgeon to find out what is important to you. During decision-making, it is important to establish with your doctor/surgeon **'what matters to you'.**

If we know some things about you it will allow us to get to know you as a person, rather than just as a patient, and find out what is important to you. Also, if we have information about your routine activities it allows us to adapt our care. **Please complete page 4 and page 8 and hand/ post back.**

What happens after I complete this form?

Please hand back to a member of staff or post it to us at the address on the front of this form. Our team will discuss your condition and your choices during our dedicated meeting. Your clinician will inform you of the outcome of team discussions, especially if there are further recommendations to consider.

What if I do not want to give the information?

If you don't feel like sharing the information, please inform a member of staff so we don't bother you by asking. However, if you change your mind, we will be happy to help you complete it.

Can I change the shared information once I have completed it?

Yes, we recognise that what matters to you may change during the decision-making process. For example, you may have concerns about recovery from a particular operation, but as you find out more about it, this may no longer be important. You can change what you provide at any time as it's your shared information.

Quotes from previous patients

"This is great, makes me feel that you want to take the time to know me."

"The form was easy to use and made me think about what is really important to me."

My values - What matters to me?

- Please let us know what is important to you from the list of values below.
- A member of staff can help you complete it, if you wish.
- Some things that matter to you may be physical, psychological/emotional or social.
- Or it could be something completely different. There are no 'right or wrong' answers as it is about you.

Please add a value from 0 to 10 (0 low priority, 10 high priority) next to each of the following items:

Examples of what matters to you	Importance out of 10	Top 3 (Please tick)
• Cure from leakage	0 1 2 3 4 5 6 7 8 9 10	
• Just using less pads	0 1 2 3 4 5 6 7 8 9 10	
• Avoid repeat surgery in the future	0 1 2 3 4 5 6 7 8 9 10	
• Undergoing day surgery	0 1 2 3 4 5 6 7 8 9 10	
• Shorter hospital stay	0 1 2 3 4 5 6 7 8 9 10	
• Quick recovery and quick return to normal activities	0 1 2 3 4 5 6 7 8 9 10	
• Avoid major abdominal surgery	0 1 2 3 4 5 6 7 8 9 10	
• Avoid future surgery for prolapse	0 1 2 3 4 5 6 7 8 9 10	
• Least pain after surgery	0 1 2 3 4 5 6 7 8 9 10	
• Avoiding mesh complications	0 1 2 3 4 5 6 7 8 9 10	
• Avoiding self-catheterisation	0 1 2 3 4 5 6 7 8 9 10	
• Avoid general anaesthesia	0 1 2 3 4 5 6 7 8 9 10	
• Avoid local anaesthesia	0 1 2 3 4 5 6 7 8 9 10	
• Other ___________________	0 1 2 3 4 5 6 7 8 9 10	

Other examples to consider

Physical

- How I prefer to have surgery – for example, which anaesthetic / pain relief.
- I find it difficult to be awake during surgery.
- I am concerned about foreign materials left permanently inside my body.
- I do not want to stay overnight in hospital.

Psychological/emotional

- The information I need is in a way that I understand.
- I feel isolated in the room on my own and need staff to check in on me regularly.
- I am not good at tolerating pain at all and get distressed quite quickly. It is important for me to get pain relief on time.
- I live a long way from the hospital and am not able to return for repeat treatment. It is important for me to receive treatment that works in the long-term.

Social

- I would like my family (daughter) to be involved in the decision.
- My elderly husband will need to be in respite care while I am in hospital. It is important that I involve him in this decision too.

My non-surgical options

Reminder of the Management Pathway for Women with Stress Urinary Incontinence (SUI).

- Women with **bothersome** leakage on coughing, sneezing or physical activities wishing treatment.

↓

Lifestyle advice

- Review of dietary and fluid intake.
- Weight loss programme for women with increased weight.
- Referral to continence nurse for bladder training, fluid management and further advice.

↓

Pelvic floor muscle training (PFMT)

- All women with SUI should be offered a PFMT programme by an appropriately trained specialist, usually a physiotherapist.
- PFMT can also be useful in women with mixed symptoms – for example, urgency.

↓

Medical treatment

- Some women may consider a trial of Duloxetine therapy.
- Potential side-effects and duration of treatment should be considered.

↓

Vaginal devices

- Some women may consider a continence pessary or device if suitable.

↓

You are here

Surgery for women with SUI*

- Surgery should only be considered if the above treatment options have not improved symptoms.
- Patient refusal of any of the above treatment option should be documented.
- Individual patient condition needs to be discussed by the continence team.

* Please ask your doctor for the specific leaflet about the treatment(s) you are considering.

Referral to a different clinician (or a different hospital) may be required, depending on availability of surgical procedures.

My surgical options

Table comparing the main advantages and disadvantages of the four surgical procedures for treatment of stress urinary incontinence in women.

Procedure	Main advantages	Main disadvantages	What if it does not work?
**Mesh tape**<br>Surgery where a piece of plastic mesh tape is inserted to support the urethra (tube that carries urine from the bladder to outside the body).	• Day surgery<br>• Quick recovery<br>• The standard procedure since 2000	• Mesh complications (can cause long-term pain and may require surgical removal)<br>• Long-term risks remain unknown	• Repeat surgery carries increased risks and technical difficulties<br>• Repeat surgery may be less successful
**Colposuspension**<br>Surgery where the neck of the bladder is lifted upwards and stitched in place.	• Avoids mesh complications<br>• The standard procedure before 2000<br>• Can be done by keyhole surgery (some units)	• Major abdominal surgery<br>• Risk of future pelvic organ prolapse (may require surgery)	
**Natural tissue sling**<br>Surgery where a sling of your own tissues is inserted around the neck of the bladder to support it.	• Avoids mesh complications<br>• Higher cure and improvement rate	• Major abdominal surgery<br>• Higher risk of difficulty emptying the bladder (need for self-catheterisation)	
**Urethral bulking agents**<br>Surgery where a substance is injected into the walls of the urethra to increase its size and allow it to remain closed with more force.	• Avoids mesh complications<br>• Day surgery (usually local anaesthesia)<br>• Least invasive as no skin cuts	• Short-term success compared to other surgical procedures<br>• Repeated injections may be required – no reliable evidence on long-term success	• Repeat surgery is safest<br>• No impact on success of future surgery

My choice (please complete this table)

Procedure	I will choose this option because…	I will NOT choose this option because…
Mesh tape		
Colposuspension		
Natural tissue sling		
Urethral bulking agent injection		

Patient's signature: ....

Patient name: ....

Date: ....

Please write any further comments here: ....

....

....

....

Your notes

FOR OFFICE USE ONLY

**Procedure**	**Outcome of MDT discussion**<br><br>**Date:**	**Outcome of further patient consultation if necessary**<br><br>**Date:**
Mesh tape		
Colposuspension		
Natural tissue sling		
Urethral bulking agent injection		

Clinician signature: ....

Clinician name: ....

Date: ....

All of our publications are available in different languages, larger print, braille (English only), audio tape or another format of your choice.

Wszystkie nasze publikacje są dostępne w różnych językach, dużym drukiem, brajlem (tylko w wersji angielskiej), na taśmie dźwiękowej lub w innym formacie Twojego wyboru.

我們所有的印刷品均有不同語言版本、大字體版本、盲文（僅有英文）、錄音帶版本或你想要的另外形式供選擇。

كافة مطبوعاتنا متاحة بلغات مختلفة و بالأحرف الطباعية الكبيرة و بطريقة بريل الخاصة بالمكفوفين (باللغة الإنكليزية فقط) و على شريط كاسيت سمعي أو بصيغة بديلة حسب خيارك.

Tha gach sgrìobhainn againn rim faotainn ann an diofar chànanan, clò nas motha, Braille (Beurla a-mhàin), teip claistinn no riochd eile a tha sibh airson a thaghadh.

हमारे सब प्रकाशन अनेक भाषाओं, बड़े अक्षरों की छपाई, ब्रेल (केवल अंग्रेज़ी), सुनने वाली कसेट या आपकी पसंदनुसार किसी अन्य फ़ॉरमेट (आरूप) में भी उपलब्ध हैं।

我们所有的印刷品均有不同语言版本、大字体版本、盲文（仅有英文）、录音带版本或你想要的另外形式供选择。

ہماری تمام مطبوعات مختلف زبانوں، بڑے حروف کی چھپائی، بریل (صرف انگریزی)، سننے والی کسٹ یا آپ کی پسند کے مطابق کسی دیگر صورت (فارمیٹ) میں بھی دستیاب ہیں۔

ਸਾਡੇ ਸਾਰੇ ਪਰਚੇ ਅਤੇ ਕਿਤਾਬਚੇ ਵਗ਼ੈਰਾ ਵੱਖ ਵੱਖ ਭਾਸ਼ਾਵਾਂ ਵਿਚ, ਵੱਡੇ ਅੱਖਰਾਂ ਅਤੇ ਬ੍ਰੇਲ (ਸਿਰਫ਼ ਅੰਗਰੇਜ਼ੀ) ਵਿਚ, ਆੱਡੀਓ ਟੇਪ 'ਤੇ ਜਾਂ ਤੁਹਾਡੀ ਮਰਜ਼ੀ ਅਨੁਸਾਰ ਕਿਸੇ ਹੋਰ ਰੂਪ ਵਿਚ ਵੀ ਮਿਲ ਸਕਦੇ ਹਨ।

☎ 0800 169 1441

reviewed for plain English

Tell us what you think...

If you would like to comment on any issues raised by this document, please complete this form and return it to: Communications Department, 28 Lister Street, Crosshouse Hospital, Crosshouse KA2 0BB. You can also email us at: comms@aaaht.scot.nhs.uk.

If you provide your contact details, we will acknowledge your comments and pass them to the appropriate departments for a response.

Name ______________________________

Address ______________________________

____

Comment ______________________________

____

Last reviewed: November 2017
Leaflet reference: MIS17-214-GD
